# Effects of propofol on macrophage activation and function in diseases

**DOI:** 10.3389/fphar.2022.964771

**Published:** 2022-08-17

**Authors:** Shuyuan Yi, Xinyi Tao, Yin Wang, Qianqian Cao, Zhixia Zhou, Shoushi Wang

**Affiliations:** ^1^ School of Anesthesiology, Weifang Medical University, Weifang, China; ^2^ Institute for Translational Medicine, The Affiliated Hospital of Qingdao University, College of Medicine, Qingdao University, Qingdao, China; ^3^ Qingdao Central Hospital, Central Hospital Affiliated to Qingdao University, Qingdao, China

**Keywords:** macrophage, propofol, inflammation, tissue repair, tumor

## Abstract

Macrophages work with monocytes and dendritic cells to form a monocyte immune system, which constitutes a powerful cornerstone of the immune system with their powerful antigen presentation and phagocytosis. Macrophages play an essential role in infection, inflammation, tumors and other pathological conditions, but these cells also have non-immune functions, such as regulating lipid metabolism and maintaining homeostasis. Propofol is a commonly used intravenous anesthetic in the clinic. Propofol has sedative, hypnotic, anti-inflammatory and anti-oxidation effects, and it participates in the body’s immunity. The regulation of propofol on immune cells, especially macrophages, has a profound effect on the occurrence and development of human diseases. We summarized the effects of propofol on macrophage migration, recruitment, differentiation, polarization, and pyroptosis, and the regulation of these propofol-regulated macrophage functions in inflammation, infection, tumor, and organ reperfusion injury. The influence of propofol on pathology and prognosis via macrophage regulation is also discussed. A better understanding of the effects of propofol on macrophage activation and function in human diseases will provide a new strategy for the application of clinical narcotic drugs and the treatment of diseases.

## 1 Introduction

Propofol (2,6-diisopropylphenol) is a gamma-aminobutyric acid (GABA) receptor agonist and an intravenous anesthetic that is commonly used in the clinic. It is often used in the induction and maintenance of anesthesia. Propofol induction is rapid and stable with almost no excitation and a low incidence of postoperative nausea and vomiting (PONV) (Gao et al., 2020). Based on these advantages, propofol is suitable for sedation and anesthesia in most types of surgery. Propofol also regulates the inflammatory response (Ma et al., 2013; Li et al., 2015) and exerts antioxidant effects (Tanaka et al., 2010). Increasing evidence has shown that the use of propofol also affects the development of tumors and has long-term implications for patient outcomes (Zhang et al., 2014; Cata et al., 2020). Increasing attention has been given to its possible mechanism. Propofol reduces the phagocytic activity of neutrophils in innate immunity and inhibits the release of pro-inflammatory cytokines, such as interleukin-6 (IL-6) and tumor necrosis factor-α (TNF-α), from peripheral blood mononuclear cells (Visvabharathy and Freitag, 2017). The regulation of propofol on the function of immune cells, including lymphocytes, neutrophils, natural killer (NK) cells and macrophages, and the potential mechanisms have been the focus of research in recent years.

Macrophages originate from progenitor cells in the bone marrow and enter the bloodstream, where monocytes migrate to tissues and differentiate into macrophages during inflammation or cancer (Gordon and Martinez, 2010). Macrophages are highly plastic immune cells that are involved in regulating the inflammatory response, secreting cytokines, removing cell fragments, killing pathogens, and maintaining tissue development and homeostasis (Varol et al., 2015). Macrophages play an important role in the development of inflammatory diseases, including diabetes (Meshkani and Vakili, 2016), infection (Kotani et al., 2001), atherosclerosis (Moore et al., 2013), reperfusion injury (Sousa et al., 2021), and cancer (Vitale et al., 2019; Wu et al., 2020). Depending on the local environment, macrophages differentiate into a variety of phenotypes and secrete a variety of inflammatory cytokines, which act as pro-inflammatory or anti-inflammatory agents. Increasing evidence shows that the inflammatory or anti-inflammatory response involved in macrophages is also regulated by perioperative anesthetics, which in turn affects the development and treatment of the disease. Dexmedetomidine and propofol inhibit the expression of lipopolysaccharide (LPS)-stimulated high mobility group box 1 (HMGB1) in macrophages (Chang et al., 2013; Jia et al., 2017). The volatile anesthetic sevoflurane modulates macrophage recruitment, polarization, differentiation, apoptosis and pyroptosis by regulating the caspase-1, reactive oxygen species (ROS), forkhead box O1 (FoxO1), p21, and glycogen synthase kinase-3 (GSK-3β)/nuclear factor-E2-related factor 2 (Nrf2) pathways, which are involved in local immunity and tumor growth (Jin et al., 2013; Fu et al., 2020; Sztwiertnia et al., 2020; Cai et al., 2021a). Opioids, such as morphine, also inhibit the phagocytosis of macrophages and are associated with the reduction of macrophage colony-stimulating factor-induced proliferation and differentiation (Sacerdote et al., 2003). Remifentanil increases osteoclasts by enhancing migration and cell fusion of bone marrow-derived macrophages (BMM_S_) (Jeon et al., 2018). Therefore, the activation, polarization and function of macrophages are regulated by a variety of anesthetics during the perioperative period, especially propofol. Notably, increasing attention has been given to the prominent anti-inflammatory effect of propofol and its regulation of macrophage function, which reduce inflammation and reperfusion injury and improve tumor prognosis.

Therefore, a better understanding of the regulation of macrophage activation and function mediated by anesthetics and its consequent effects on the immune system and disease development will provide new strategies for clinical perioperative anesthetics and disease treatment. In this review, we summarize the effects of propofol on macrophage activation, including migration, recruitment, differentiation, polarization, and pyroptosis. Moreover, based on recent preclinical and clinical studies, the function of these propofol-related macrophages in local inflammation, infection, tumor, and tissue repair was highlighted. This review will enrich the theoretical basis of the research of anesthetics in human disease development and treatment.

## 2 Macrophage activation and function

Macrophages are one of the most important parts of the mononuclear phagocyte system (van Furth et al., 1972). There are two distinct origins of macrophages in tissues. Most macrophages in healthy tissues are established before birth. The other origin is associated with pathology, homeostasis, and tissue inflammation, where macrophages develop from tissue-infiltrating monocytes in adulthood (Varol et al., 2015). The CD115^+^ mouse monocyte subsets are divided into Ly6C^hi^ monocytes and Ly6C^low^ monocytes (Ingersoll et al., 2010). The primary function of Ly6C^low^ cells appears to involve surveying vascular endothelial integrity and involvement in early inflammation and tissue repair responses. In contrast, Ly6C^hi^ monocytes in mice and CD14^+^ monocytes in humans serve as precursors to tissue macrophages derived from monocytes and are recruited to pathological changes, such as tumors and inflammation, to exert pro-inflammatory and antibacterial activities (Auffray et al., 2007; Carlin et al., 2013). Under the stimulation of the microenvironment and cytokines, Ly6C^hi^ cells enter damaged tissues and differentiate into macrophages to reduce inflammation and promote tissue repair by stimulating angiogenesis, suppressing neutrophil aggregation and removing cellular debris (Varol et al., 2015). Depending on the difference in anatomical locations and functional phenotypes, tissue-resident macrophages are divided into microglia, osteoclasts, alveolar macrophages, and Kupffer cells (Davies et al., 2013).

The activation and polarization of macrophages is always influenced by the surrounding microenvironments in inflammatory or tumor environments. Macrophages generally polarized into two subtypes, the classically activated type (M1) and the alternating activated type (M2). M1 macrophages are generally induced by Th1-type cytokines, such as interferon-γ (IFN-γ) and TNF-α, or recognized by bacterial LPS. These macrophages produce and secrete higher levels of proinflammatory cytokines, such as TNF-α, IL-1β, IL-6 and cyclooxygenase 2 (COX-2). Therefore, M1 macrophages have strong antigenic presentation, antimicrobial and antitumor activities, which also mediate ROS-induced tissue injury and promote tissue regeneration or wound healing. The Th2 cytokines IL-4 and IL-13 induce the activation of M2 macrophages and further induce the production of different surface receptors and effector molecules, such as IL-10 and TGF-β. M2 macrophages have a strong phagocytic capacity and remove debris and apoptotic cells, promote tissue repair and wound healing, and promote angiogenesis and fibrosis (Shapouri-Moghaddam et al., 2018; Wu et al., 2020). The exposure of M2 macrophages to M1 signals or the exposure of M1 macrophages to M2 signals induced the repolarization or rearrangement of differentiated macrophages. Therefore, the function of macrophages is usually depend on their polarization state.

**FIGURE 1:**
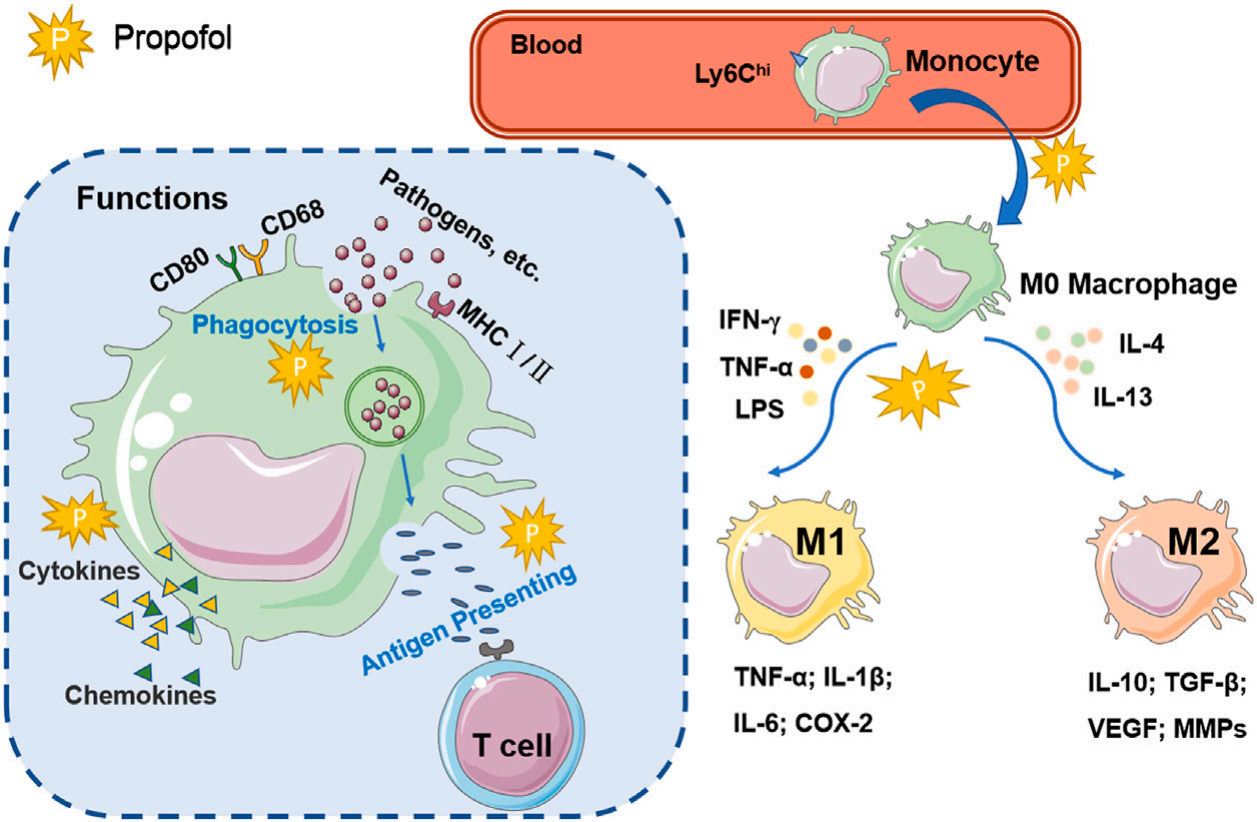
The activation and function of macrophages. Ly6C^hi^ monocytes in the blood enter the damaged tissue and differentiate into macrophages. Macrophages recruited by inflammation or tumors are stimulated by Th1 or Th2 cytokines to differentiate into two activated types: the classical activated type (M1) and the alternating activated type (M2). Activated macrophages express high levels of CD80, CD68, and MHC I/II. Macrophages phagocytize pathogens and cell fragments, recognize specific antigens, and transmit signals to T cells for antigen presentation. Macrophages participate in immune regulation by regulating the secretion of cytokines and chemokines. The explosion-shaped graphic represents propofol, suggesting that it may be involved in the signaling pathways and cellular processes of macrophages.

As shown in Figure 1, the functional characteristics of macrophages are related to changes in the tissue microenvironment and cytokines. Macrophages play a role in immune surveillance via several receptors, including scavenger receptors, cytokine receptors, adhesion molecules, nuclear hormones and pattern recognition receptors, such as Toll-like receptors (TLRs) and Nod-like receptors (Varol et al., 2015). Activated macrophages highly express antigen-presenting molecules on the surface, such as CD80, CD68 and MHC class Ⅰ and Ⅱ molecules. As important antigen-presenting cells (APCs), macrophages play the role of antigen presentation by recognizing and phagocytizing pathogens and stimulating T cells (Wynn et al., 2013). During inflammation or infection, macrophage apoptosis is another strategy used by the host to limit infection and defend against pathogens, and it is an important process in the development of diseases associated with reduced pathogen activity. Macrophage apoptosis is associated with a reduction in lesion size in arteriosclerosis (Shapouri-Moghaddam et al., 2018). If the pathogen inhibits macrophage apoptosis and promotes necrosis, antigen presentation by macrophages may be impaired, and immune escape may occur (Moraco and Kornfeld, 2014). Macrophages are involved in the induction of adhesion molecules, pro-angiogenic factors, matrix metalloproteinase (MMP), nuclear factor-kappa B (NF-κB) and signal transducers and activators of transcription-1 (STAT1) via the regulation of interleukins (Wu et al., 2020). In conclusion, as a non-specific immune cell, macrophages exert phagocytic properties to directly engulf and break down cancer cells, antigens, senescent damaged cells and bacteria. Macrophages can take up and process antigens and deliver information to T lymphocytes. In addition, the ability to secrete bio-active mediators makes macrophages important cells involved in the inflammatory response.

## 3 Macrophages and anesthetic

A variety of anesthetics participate in macrophage activation, polarization, and functional regulation. Although the effects of some anesthetics are controversial, it is well known that they influence the development and prognosis of disease by regulating macrophages.

*In vivo* animal models constructed with toxins and tumors showed that anesthetics and techniques had different effects on the progression of inflammation and tumor prognosis by regulating macrophage activation and function. Clinical histological studies showed that the distribution of multiple immune cells, especially macrophages, had the potential to predict prognosis and treatment in inflammatory sites and tumor tissues. Tissue damage and the use of opioids may activate neuroglia, and the early activation of microglia and macrophages after surgery or exposure to remifentanil may be related to the immune response to injury (Romero et al., 2013). Peroxisome proliferator-activated receptor gamma (PPARγ) negatively regulated macrophage activation. Dexmedetomidine also promotes the M2 activity of macrophages via the PPARγ/STAT3 pathway and inhibits the pro-inflammatory innate immune activation in the liver (Zhou et al., 2020). Dexmedetomidine also modulates the activation of Kupffer cells by activating alpha 2-adrenergic receptors (α2B-ARs), which transforms them into anti-inflammatory phenotypes and promotes liver regeneration (Yue et al., 2022).

Receptors, signaling pathways and transcription factors also influence macrophage polarization. Sevoflurane pretreatment promotes the formation of an anti-inflammatory phenotype in microglia/macrophages by up-regulating the phosphorylation of GSK-3β and Nrf2 nuclear transposition, and it has a neuroprotective effect on middle cerebral artery occlusion (MCAO) (Cai et al., 2021a). However, sevoflurane inhibits FoxO1, regulates p21 and increases the expression of TNF-α, monocyte chemotactic protein-1 (MCP-1) and IL-6, which may increase the polarization of M1 macrophages, induce a central nervous system (CNS) inflammatory response, and lead to cognitive impairment (Fu et al., 2020). This pathway suggests that inhaled anesthetics have different effects on prognosis via regulation of the process of M1-type polarization of macrophages due to different pathological conditions. Recent studies showed that electroacupuncture also prevented weight gain by regulating the inflammatory response in obese adipose tissue and promoting the polarization of M2 macrophages (Wang et al., 2021).

The volatile anesthetics isoflurane and sevoflurane inhibit macrophage phagocytosis. Sevoflurane binds directly to the Ras-like small GTPase RAP1 and inhibits its activity. Isoflurane may interact with molecules in the RAP1 cascades by inhibiting macrophage-1 antigen (Zha et al., 2019). Notably, another study demonstrated that sevoflurane upregulated inducible nitric oxide synthase (iNOS), inhibited the NF-κB pathway, enhanced the LPS-stimulated phagocytosis of macrophages on bacteria, and improved the bactericidal and anti-inflammatory mechanisms of endotoxemia (Gerber et al., 2019). This difference may be due to the different physical and chemical properties of macrophages under different stimuli and the multiple pathways of volatile anesthetic regulation of macrophages. Opioid analgesics also promoted macrophage apoptosis and reduced the expression of toll-like receptor 4 (TLR4) on macrophages (Franchi et al., 2012; Kim, 2018; Lin et al., 2021). Another intravenous anesthetic, ketamine, also had a significant effect on macrophage function. Nowak W et al. showed that ketamine promoted macrophage differentiation into M2 subtypes by modulating the N-methyl-D-aspartate receptor (NMDAR) and mammalian target of rapamycin (mTOR). Its prominent anti-inflammatory properties are important for the prognosis of patients with major depression and other inflammatory diseases (Nowak et al., 2019). Ketamine mediates the function of macrophages by decreasing the mitochondrial membrane potential and downregulating the activation of LPS-induced macrophages by regulating signaling pathways and transcription factors (Liu et al., 2012). However, the regulation of macrophages by anesthetics is not always beneficial to the body in disease progression. The use of sevoflurane is associated with reduced Tumor-associated macrophages (TAMs) and tumor growth in some cancer models (Sztwiertnia et al., 2020).

## 4 Propofol regulation of macrophage activation and function

Propofol, often referred to as “milk of anesthesia”, is an effective intravenous anesthetic. From the 1970s, James Baird Glen at British Chemical Industries promoted the use of propofol as a new and safe intravenous anesthetic, which was approved in the United Kingdom in 1987 and in the United States in 1989 (Walsh, 2018). Propofol can be used for induction and maintenance of general anesthesia and to enhance sedation in mechanically ventilated patients. The common pathophysiological functions of propofol mainly include inhibition of blood pressure and respiration, followed by immune system and nervous system abnormalities, and some gastrointestinal reactions, etc., which are related to the dose of propofol (Sahinovic et al., 2018). However, compared with other anesthetics, propofol induces anesthesia rapidly and steadily, and the probability of the above pathological effects is also lower (Eleveld et al., 2018). Therefore, propofol is one of the most widely used anesthetics in clinical practice.

The mechanism of action of propofol is related to the interaction of specific structures on the postsynaptic membrane. Extensive experiments have shown that inhibitory central GABAergic neurotransmission is the key to mediating the pharmacological effects of propofol (Trapani et al., 2000; Saraghi et al., 2013). GABA is an important inhibitory neurotransmitter in the central nervous system, and its effects are produced by selective interaction of GABA receptors (GABAA and GABAB) that are widely distributed in immune cells such as neutrophils, monocytes and macrophages (Bhat et al., 2010). Therefore, in addition to anesthesia, propofol also has non-anesthetic effects such as analgesia, organ protection, immunity, and anti-oxidation. Increasing attention has been given to propofol because of its outstanding anti-inflammatory and anti-oxidation characteristics. Immune cells targeted by propofol include lymphocytes, neutrophils, NK cells, and macrophages (Ji et al., 2011; Ulbrich et al., 2016; Lim et al., 2018).

**FIGURE 2:**
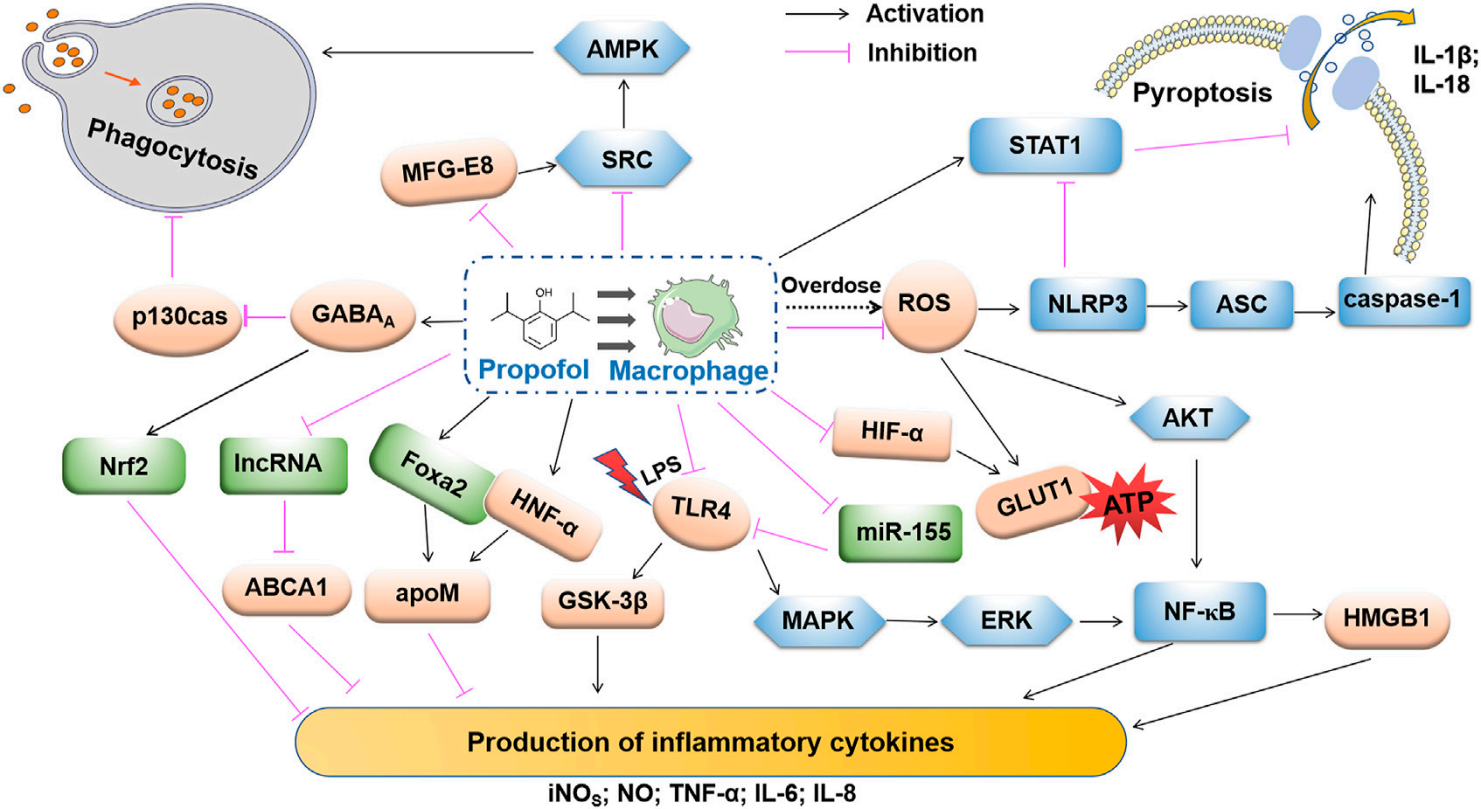
Propofol regulation in macrophage phagocytosis, pyroptosis and production of inflammatory cytokines. High-dose propofol activates pro-caspase-1 via NLRP3 and AIM2 inflammasomes via the inflammasome adaptor ASC. Propofol-induced mitochondrial ROS may trigger the activation of NLRP3. The clinical dose of propofol inhibits pyroptosis by activating STRT1. Propofol inhibits pressure effects on macrophage phagocytosis by activating the GABAA receptor and inhibiting p130cas. Propofol reduces microglial production of MFG-E8 and inhibits cellular phagocytosis using the MFG-E8-dependent SRC-AMPK pathway. Propofol inhibits the ROS-mediated AKT and NF-κB pathways. Propofol also inhibits MAPK/ERK, the upstream regulator of NF-κB. Dissociated NK-κB is transferred to the cytoplasm and further induces the release of HMGB1, which inhibits the production of pro-inflammatory factors. Propofol inhibits the release of inflammatory factors via apoM in an HNF-α- and Fox2-dependent manner. Through the expression of lncRNA LOC286367, the expression of ABCA1 inhibits the release of pro-inflammatory factors, and the inhibitory effect of propofol on LPS-activated TLR4 may be related to its down-regulation of miR-155 expression. Propofol also inhibits the expression of HIF-1α and GLUT1, which results in a decrease in ATP cytosolic accumulation and lactic acid accumulation. By regulating these signaling pathways and related molecules, propofol may play an anti-inflammatory role by inhibiting the secretion of inflammatory factors.

Macrophages widely express GABA receptors and have a mechanism for GABA catabolism (Bhat et al., 2010). Sanders et al. detected mRNAs encoding GABAA receptor α1, α2, α3, β2 and γ2 subunits in mouse macrophages (Sanders et al., 2013). Several studies have demonstrated the role of GABA receptors in the development of macrophage-associated diseases. Januzi’s team found that the expression levels of α2-type GABAA receptors were down-regulated and up-regulated respectively, during the activation of the M1 and M2 alveolar macrophage in mice (Januzi et al., 2018). For myocardial macrophages, GABAA receptors exacerbate myocardial hypertrophy and fibrosis by activating the AKT/mTOR signaling pathway and increasing the MHCII^low^/MHCII^high^ ratio in Ly6C^low^ macrophage subsets (Bu et al., 2021). GABA transporter (GAT) 2 deficiency promotes the intracellular accumulation of hypoxanthine, which not only blocks the formation of the NLRP3-ASC-caspase-1 complex, but also enhances the expression of oxidative phosphorylation related genes, affecting the production of IL-1β (Xia et al., 2021). In summary, GABA signaling is involved in the regulation of immune responses mainly by affecting macrophage activation and function, which indicated that macrophages, are key cells involved in propofol regulation of the inflammatory response and tumor immunity. Indeed, propofol affects the biological behavior of macrophages by affecting signaling pathways, gene expression, and related mediators (Figure 2). Propofol has long-term effects on inflammatory disease and tumor prognosis by regulating macrophage recruitment, differentiation, polarization, pyroptosis and functions.

### 4.1 Regulation of macrophage migration and recruitment

Tissue-resident macrophages release a variety of pro-inflammatory mediators to facilitate circulation and macrophage recruitment in monocytes. Monocytes and macrophages migrate to the site of inflammatory injury to eliminate inflammatory signals and promote wound healing and tissue repair (Shapouri-Moghaddam et al., 2018). Intraoperative hyperoxia leads to the aggregation of macrophages, as represented by an increased expression of IL-8. IL-8 and TNF-α are effective chemokines involved in the regulation of propofol on macrophages (Jia et al., 2017).

The integrity of mitochondrial function is the key to maintaining the chemotaxis, migration and phagocytosis of macrophages. Clinically relevant concentrations of propofol (3 and 30 μm) may inhibit macrophage function by inhibiting mitochondrial membrane potential and adenosine triphosphate (ATP) synthesis rather than exert direct cytotoxicity (Chen et al., 2003). Wu et al. also found that propofol reduced macrophage ATP biosynthesis and migration by inhibiting mitochondrial membrane potential (Wu et al., 2005). Mitochondrial dysfunction may be a key cause of propofol-induced immunosuppression of macrophages. Therefore, the inhibitory effect of propofol on macrophage migration and recruitment may be a new target in the treatment of macrophage-associated inflammatory diseases and tumors.

### 4.2 Regulation of macrophage differentiation and polarization

Macrophages migrate to the site of local injury and infection, which results in local and systemic acute and chronic inflammation. Phagocytosis, antibacterial activity, and cytotoxicity are enhanced, which contribute to initiating repair and resolving inflammation and tumor cells. Although propofol does not affect the recruitment of blood monocytes into the kidney in *Staphylococcus aureus* infection, it may prevent monocytes from differentiating into macrophages and expanding the inflammatory abscesses in the kidney (Visvabharathy and Freitag, 2017).

Propofol affects epigenetic pathways, such as long noncoding RNAs (lncRNAs), microRNAs (miRNAs), and histone acetylation, and it modulates genetic signaling pathways, including hypoxia, ROS, NF-κB, mitogen-activated protein kinase (MAPK), and Nrf2. Notably, propofol also affects tumor immune function and the degree of immunosuppression (Jawan et al., 2008; Ferrando et al., 2013; Ma et al., 2018; Gao et al., 2021). MiRNAs regulate genes at the transcriptional level and induce gene silencing, which play an important role in many aspects of macrophage biology, including influencing macrophage biology and pathological conditions. Propofol regulates the number and biological activity of macrophages by regulating miRNAs at the transcriptional level. MiR-142-3p exerts antitumor effects by regulating the differentiation of macrophages. However, propofol exerts antitumor activity by regulating the expression of miR-142-3p in macrophage-derived microvesicles (Zhang et al., 2014).

**FIGURE 3:**
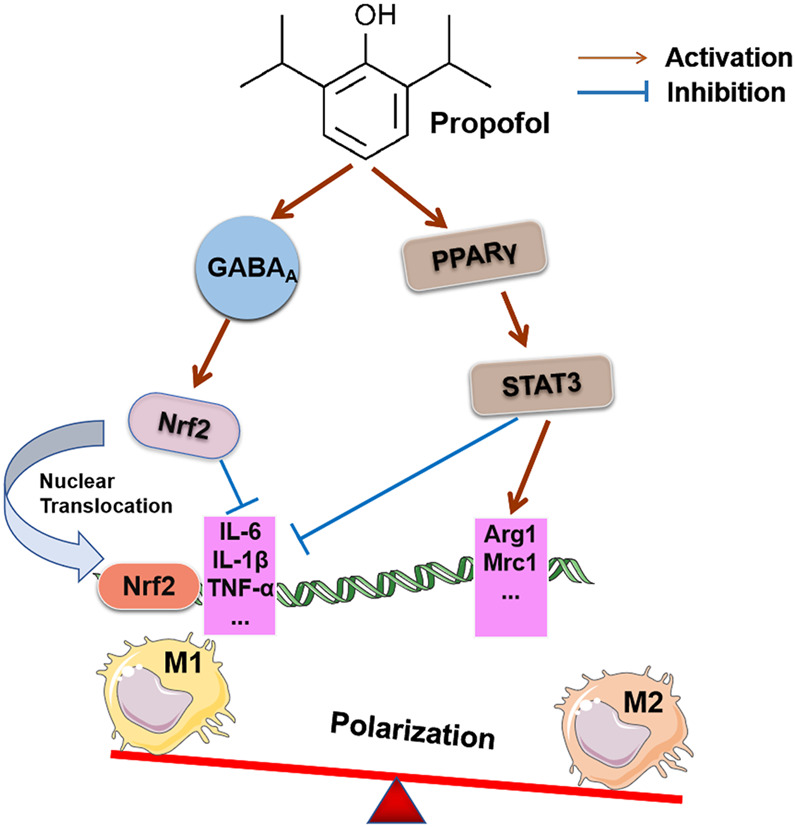
Effects of propofol on macrophage polarization. Invasive surgery may cause M1 macrophage polarization and related complications. Propofol inhibits the expression of IL-6 and IL-1β by activating the GABAA receptor to induce Nrf2 accumulation in the cytoplasm and its translocation into the nucleus, which prevents the inflammatory response during the polarization of human macrophages. Propofol treatment stimulated PPARγ activation in the rI/R model and enhanced PPARγ-mediated STAT3, decreased iNO_S_ mRNA of the M1 target gene, increased Mrc1 and Arg1 mRNA expression of the M2 target gene, and promoted M2 polarization in macrophages.

Current studies show that propofol exposure to human monocytic leukemia THP-1 cells induces Nrf2 cytoplasmic accumulation and nuclear translocation by activating GABAA receptors, which inhibits the inflammatory response during the polarization of M1 macrophages and down-regulates the expression of IL-6 and IL-1β. However, it did not affect the function of M2 macrophages or change the gene expression of M2 markers [e.g., IL-10, transforming growth factor-(TGF-) β, and CD206]. The reason may be that propofol does not alter the anti-inflammatory or tissue repair functions of M2 macrophages (Kochiyama et al., 2019). However, a recent study suggested that propofol improved renal ischemia-reperfusion injury by inducing the expression of the M2 polarization marker via the PPARγ/STAT3 pathway (Liu et al., 2021b). The pathways and effect of propofol on the polarization of macrophages are shown in Figure 3. This result suggests that the degree of macrophage differentiation or the underlying state of the disease influences the effect of propofol on the classification and polarization of macrophages. Under pathological conditions, propofol may modulate the polarization of macrophages in different directions, which is influenced by local inflammatory signaling pathways, cytokines secreted by immune cells and the microenvironment. However, there are few studies on the role of propofol in the polarization of macrophages, and its effect on the polarization of macrophages and its mechanism must be further studied.

### 4.3 Regulation of macrophage pyroptosis

Propofol inhibited macrophage phagocytosis, chemotaxis, and oxidative burst in the model of propofol infusion syndrome (PRIS) (Wheeler et al., 2011; Hsing et al., 2012; Sun et al., 2019), but the mechanism of this immune disorder is not clear. Exposure of macrophages to propofol may inhibit phagocytosis and apoptosis. Pyroptosis is a specific form of apoptosis that is involved in immune regulation and is caused by inflammatory bodies. Mitochondrial dysfunction induced specific activation of NLRP3-ASC inflammatory corpuscles, and propofol significantly induced mitochondrial dysfunction. High-dose propofol induced mitochondrial apoptosis and caspase-1-dependent pyroptosis in macrophages. Propofol-induced pyroptosis triggers macrophage death and mediates the release of IL-1β and IL-18 (Sun et al., 2019). However, Liu et al. found that a clinical dose of propofol inhibited the activation of IL-1β and IL-18 in rat alveolar macrophages or lung tissue and inhibited the activation of inflammatory bodies and pyroptosis by up-regulating sirtuin-1 (STRT1) in the lung, which attenuated acute lung injury caused by renal reperfusion injury (Liu et al., 2020). These results suggest that the regulatory effect of propofol on the focal death of macrophages may depend on the administration dose and pathological state of the body. Therefore, elucidating the mechanism of propofol-induced macrophage pyroptosis will further deepen our understanding of propofol-induced immune dysfunction.

### 4.4 Propofol regulation of macrophage function

**FIGURE 4:**
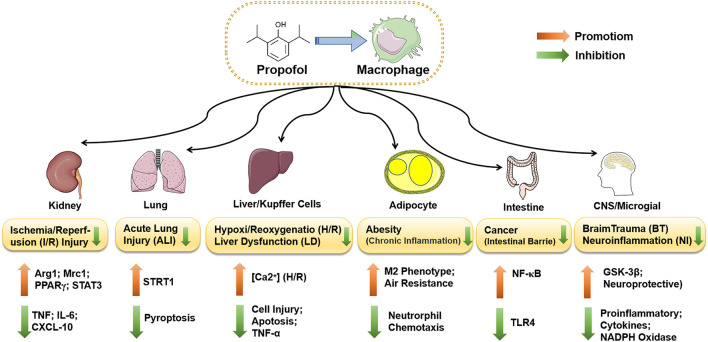
The functions of propofol-regulated macrophages in different diseases. Propofol suppresses the expression of STRT1 by inhibiting IL-1β and IL-18 in rat alveolar macrophages or lung tissue, which inhibits cell pyroptosis and attenuates ALI induced by rl/R. Propofol inhibits the activation of caspase-3 and the apoptosis of hepatocytes and alleviates LPS-induced liver dysfunction. During H/R, the inhibitory effect may be induced by the increase in [Ca2^+^]i in Kupffer cells. Propofol also inhibits the TLR/NF-ΚB pathway via microRNA, reduces the release of inflammatory factors, and protects the intestinal mucosal barrier of colorectal cancer. Propofol partially protects microglia by downregulating TLR4 and inducing inactivation of GSK-3β, which inhibits the release of inflammatory factors and reduces LPS-induced neuroinflammation (NI). Neuroprotection after brain trauma (BT) may result from propofol reducing the expression of two key components of NADPH oxidase, P22phox and gp91phox.

Although macrophages effectively control infection, remove necrotic tissue and promote tissue repair and wound healing, they also cause tissue damage and pathological changes during infection and inflammatory disease. Several studies demonstrated that propofol participated in the regulation of macrophage function, regulated the energy metabolism of macrophages during inflammation and infection, altered the activated state of macrophages, and induced phagocytosis. As important immune cells, macrophages are not independent of their functional properties and effects on health or disease but are multidirectional and interconnected with each other. The effect of propofol on the function of macrophages has a significant impact on disease progression. We discuss the effects of propofol on human disease by regulating macrophages for inflammation, infection, and tumor and tissue repair. The potential anti-inflammatory effect of propofol on the disease of different organs or cells by mediating macrophages is summarized in Figure 4.

#### 4.4.1 Inflammation

Mononuclear phagocytes are the host’s first line of defense against infection and inflammation and play a vital role in wound repair. The mechanism of propofol on inflammation is not clear. However, propofol also inhibits macrophage function and reduces pro-inflammatory cytokine release under certain conditions (Chen et al., 2005). Propofol alleviates pulmonary inflammation in a respiratory distress model (Haitsma et al., 2009) and reduces endotoxin inflammation by inhibiting the production of ROS and downstream protein kinase B (AKT)/inhibitor of κB kinase (IKK β)/NF-κB signaling (Hsing et al., 2011). It also inhibits the activation of Kupffer cells and regulates the expression of NF-κB and P65 protein to alleviate LPS-induced inflammation and liver cell damage (Sung et al., 2005; Li et al., 2015; Li et al., 2016). Propofol inhibited acute local inflammation in the carrageenan-induced air pouch infection model by reducing the secretion of proinflammatory cytokines (TNF-α and IL-6) and neutrophil chemokines in intra-pouch secretion (Inada and Kamibayashi, 2021). However, propofol does not affect LPS-induced NO and TNF-α in mixed glial cells (Shibakawa et al., 2005), which suggests that the effect of propofol on inflammation may be related to the differentiation and expression pattern of macrophages.

LPS is an ingredient in the outer membrane of Gram-negative bacteria, and the activation of nuclear transcription factor NF-κB and activator protein-1 (AP-1) by TLR-4 on the surface of macrophages induced the release of a large number of inflammatory and cytokines, including IL-6, IL-8, and TNF-α (Jia et al., 2017). The inhibitory effect of propofol at clinically relevant concentrations on LPS-activated TNF-α may be related to the inhibition of NF-κB and TLR-4 expression (Wu et al., 2009). In conclusion, the anti-inflammatory and anti-oxidative effects of propofol are primarily mediated by its regulation of the biosynthesis of cytokines (e.g., TNF-α, TNF-β, IL-1, IL-6, and IL-10).

Propofol also inhibited nicotinamide adenine dinucleotide phosphate (NADPH) oxidase-mediated proinflammatory cytokine production. Long-chain non-coding RNALOC286367 is involved in propofol-induced inhibition of IL-6, TNF-α and IL-1β expression in THP-1 cells (Ma et al., 2018). *In vitro* experiments and many clinical studies observed the positive effect of propofol on the reduction of inflammation. Previous clinical trials demonstrated that the inhaled anesthetic and intravenous anesthetic propofol had a positive effect on proinflammatory cytokine gene expression. A clinical study compared gene expression changes in alveolar macrophages during general anesthesia with propofol and isoflurane. The gene expression of IL-8 and IFN-γ increased after 4 h of propofol anesthesia. IL-8 is an effective chemokine, and high expression of the IL-8 gene and intraoperative hyperoxia exposure stimulate macrophage aggregation and enhance alveolar macrophage antibacterial and immune responses (Kotani et al., 2000). The anti-inflammatory effect of propofol in THP-1 cells may be mediated by apolipoprotein M (apoM) in a manner dependent on hepatocyte nuclear factor- (HNF-)1α (Ma et al., 2013). Prostaglandins are abundant in areas of trauma and inflammation. Prostaglandins are produced by COX and specific prostaglandin synthetase. Prostaglandin synthesis in macrophages primarily involves prostaglandin E2 (PGE_2_) and thromboxane (TX) A_2_ (Smith et al., 2000; Inada et al., 2010). PGE_1_ reduces alveolar macrophage aggregation during anesthesia, possibly because it inhibits the expression of CD11A, CD18, CD54 and other adhesion molecules (Kotani et al., 2001). Propofol inhibits the production of PGE_2_ in human peripheral blood mononuclear cells (Kambara et al., 2009) and peritoneal macrophages (Inada et al., 2010) by inhibiting COX-2 activity and promotes the production of IFN-γ by other immune cells, such as NK cells, to regulate inflammation and immune function.

#### 4.4.2 Infection

Perioperative infections, such as surgical site infections and pneumonia, significantly increase mortality and economic burden. Although some *in vitro* studies showed that propofol decreased the secretion of proinflammatory cytokines and had an active anti-inflammatory effect, it may have a negative effect on the regulation of immune cells in a model of microbial infection. Lavanya. V et al. demonstrated that short-term exposure to propofol also significantly increased host susceptibility to microbial infection (Visvabharathy et al., 2015). Propofol is commonly used to sedate and anesthetize intensive care unit (ICU) patients, and *Staphylococcus aureus* infection is a common complication of nosocomial infection in long-term hospitalization or ICU patients (Pofahl et al., 2011). *Staphylococcus aureus* infection causes skin infection, severe sepsis and other clinical-pathological changes, and macrophages play an important role in the control of these infections. Previous studies showed that propofol inhibited a variety of functions of macrophages, including chemotaxis, phagocytosis, and ROS production. When *Staphylococcus* aureus-infected RAW264.7 cells were exposed to propofol, IL-1β secretion and ROS levels were significantly decreased, which increased bacterial survival, but propofol did not affect the expression of IL-1β mRNA (Chen et al., 2018). This inhibitory effect on the phagocytosis of RAW264.7 cells was also mediated by lipofundin in propofol (Chen et al., 2020). However, previous studies showed that propofol reduced the LPS-induced levels of IL-1β, IL-6, TNF-α mRNA, and corresponding proteins at the pre-translation level (Chen et al., 2005). Propofol did not reduce the *in vitro* phagocytosis of *E. coli* by macrophages (Zha et al., 2019). This difference may be due to the different regulatory mechanisms of stimulating and proinflammatory cytokines. *In vivo* studies found that propofol anesthesia significantly increased the bacterial burden and renal pathology in mice infected with *Staphylococcus aureus*. This effect may be due to a reduction in the number of mature phagocytes in the kidney and increased dissemination of bacteria in the kidney tissue (Visvabharathy and Freitag, 2017). The effect of propofol on the phagocytic bacterial microbiome of macrophages strongly correlated with the type of infecting pathogen.

Mitochondria are important energy-producing organelles that participate in the activation of macrophages. Therefore, the integrity of mitochondrial function is the key to maintaining the chemotaxis, migration and phagocytosis of macrophages. Clinically relevant concentrations of propofol (3 and 30 μm) may inhibit macrophage function by decreasing mitochondrial membrane and ATP synthesis rather than direct cytotoxicity (Chen et al., 2003). Cellular ATP levels significantly correlated with macrophage function in mice with polymicrobial sepsis (Ayala and Chaudry, 1996; Chen et al., 2003). Propofol also significantly reduced the production of ATP in THP-1 cells by inhibiting LPS-induced Hypoxia inducible factor (HIF)-1 activation and the expression of the pyruvate dehydrogenase (PDH) kinase 1 (PDK-1) glycolytic process (Tanaka et al., 2010). Energy depletion of macrophages also reduces their ability to kill bacteria, which increases the risk of infection.

Studies showed that inflammation or edema increased tissue pressure, and the increase in extracellular pressure activated macrophage phagocytosis. Propofol inhibits the phagocytosis of pressure-stimulated macrophages via the GABAA receptor and p130cas phosphorylation dysregulation (Shiratsuchi et al., 2009). Propofol increases macrophage phagocytosis by inhibiting p130cas phosphorylation and reverses stress-stimulated macrophage phagocytosis mediated by GABAA receptors. Clinical studies are limited, but the incidence of postoperative pneumonia and surgical incision infection is lower with general intravenous anesthesia than volatile anesthetics in abdominal and rectal surgery (Von Dossow et al., 2007; Koo et al., 2016). It is a future target to develop new intravenous anesthetics that do not inhibit immune targets.

#### 4.4.3 Tumor development

Surgery remains one of the most effective methods to treat solid tumors. Perioperative stress and the use of anesthetics may affect the entire process of tumor development. The surgical stress response is characterized by immunosuppression, systemic inflammatory response, and excessive production of reactive oxygen species. Propofol has anti-inflammatory and antioxidative effects, and it participates in inflammatory responses by regulating immune-related cells and the subsequent production of cytokines. It is generally accepted that propofol accelerates apoptosis, promotes autophagy of cancer cells, affects the serum microenvironment, inhibits the proliferation and migration of tumor cells, and improves the malignant potential of tumors (Jaura et al., 2014; Xu et al., 2020). Lymphocytes provide direct cytotoxicity to tumor cells, and monocytes and macrophages combine directly to kill tumors and enhance endogenous immunity via antigen presentation, which makes these cells a new approach to tumor immunotherapy in the future (Anderson et al., 2021). Propofol reduced the production of vascular endothelial growth factor (VEGF) and calmodulin-dependent kinase II (CaMK II) by inhibiting NMDAR, which blocks extracellular signal-regulated kinase (ERK)/protein kinase B (AKT) signaling and ultimately reduces the malignant potential of pancreatic cancer (Chen et al., 2017). Compared to inhalation anesthesia, propofol reduced the viability of serum-cultured cells, inhibited the invasion of cancer cells, and increased cancer cell death (Kirkegaard et al., 2017). However, other studies demonstrated that the use of propofol promoted cancer cell proliferation and invasion, which increased the risk of tumor recurrence and metastasis (Zhang et al., 2012; Liu et al., 2021a). In summary, a large number of laboratory and clinical studies concluded that propofol inhibited the development of cancer in the body, but only a few results suggest that propofol promotes the progression of certain cancers. This difference may depend largely on the type and biological behavior of the tumor cells. The effect of propofol on tumor cells depends greatly on the effect of propofol on various immune cells in the tumor microenvironment (TME), especially macrophages.

Mutations caused by DNA damage are important markers of cancer. *In vitro* experiments showed that propofol at 25 μg/ml caused DNA damage in RAW264.7 cells and inhibited the mRNA expression of repair-related genes, such as DNA-dependent serine/threonine protein kinase (DNA-Pk), breast cancer gene 1 (BRCA1), O^6−^methylguanine-DNA methyltransferase (MGMT), and P50 (Wu et al., 2013). Another study showed that an overdose of propofol induced GSK-3-mediated apoptosis of lysosomes/mitochondria in macrophages (Hsing et al., 2012). The growth of malignant tumors is related to an increase in M2 cells in the TME (Najafi et al., 2019). M2 cells play a key role in tumor immune escape (Tan et al., 2018). Cytokines released by tumor cells regulate the expression of genes related to M2 angiogenesis and participate in angiogenesis. The metastasis of cancer cells may be due to the interaction with M2 cells in the TME. M2 cells release granular proteins, which act as TNF receptor inhibitors by inhibiting TNF-α binding to its receptors. TNF-α is generally released by M1 cells. As a result, the TME has a high degree of control over inflammation and tumor development. The inhibitory effect of propofol on COX-2 may also affect tumor progression because PGE_2_ may promote tumor progression by inducing bone marrow-derived suppressor cells in the TME (Sinha et al., 2007). Hypoxia significantly enhanced the pre-tumoral function of major histocompatibility complex- (MHC-)II^low^ M2-like TAMs by altering gene expression profiling rather than directly affecting TAM differentiation, which resulted in intra-tumoral heterogeneity of TAMs (Laoui et al., 2014). Under the influence of hypoxia, TAMs easily form an angiogenesis phenotype, which is involved in metabolism, angiogenesis and metastasis. HIF-1α is a key transcription factor regulating hypoxia-inducible gene expression. Hypoxia-directed TAMs promote angiogenesis by directly upregulating angiogenic molecules, such as VEGF-A, and angiogenic regulators, such as MMP7 (Henze and Mazzone, 2016). According to previous reports, propofol may inhibit the translation of HIF-1 protein via mRNA in THP-1 cells and its antioxidant activity, inhibit the accumulation of HIF-1α protein in LPS-induced macrophages (Tanaka et al., 2010). The role of macrophages and their polarization in propofol-induced tumor development has not been well studied, but the mechanism remains to be studied.

Propofol effectively inhibits the growth of hepatocellular carcinoma *in vivo* and stimulates the migration of miR-142-3p from macrophages to hepatocellular carcinoma (HCC) cells *in vitro* by down-regulating Ras-related C3 botulinum toxin substrate 1 (RAC1) and inhibiting the migration and proliferation of HCC cells (Zhang et al., 2014). Propofol also inhibits the activation of the TLR/NF-κB pathway by inhibiting the expression of miR-155, which reduces the release of inflammatory cytokines, protects the intestinal mucosal barrier and effectively inhibits bacterial translocation (Gao et al., 2021).

#### 4.4.4 Tissue repair

Notably, many clinical and preclinical studies showed that propofol reduced ischemic damage to vital organs, such as the heart (Nederlof et al., 2017), brain (Sousa et al., 2021), kidney (Liu et al., 2021b), and lung (Sousa et al., 2021). Propofol reduced CD45^+^ and increased vascular cell adhesion molecule (VCAM)-1 in the ischemic stroke model, but it did not protect the brain or lung in experimental acute ischemic stroke. Propofol showed no reduction in IL-6 and IL-1β levels in alveolar macrophages and endothelial cells in this model (Sousa et al., 2021). During organ reperfusion, activation of the endothelium promotes the adhesion and infiltration of immune cells, such as lymphocytes, macrophages, dendritic cells, and neutrophils. The protective effect of propofol on reperfusion organs and tissue repair may be mediated by inhibiting the release of inflammatory mediators, such as TNF-α, IL-6 and IL-1β. Activated macrophages, neutrophils and lymphocytes release pro-inflammatory mediators that trigger inflammation and lead to dysfunction and tissue damage (Kezić et al., 2017). Peroxisome proliferator-activated receptor (PPAR)-γ is a member of the nuclear receptor PPAR subfamily and is widely expressed in many tissues (Singh et al., 2019). PPARγ also plays an important role in organ ischemia–reperfusion injury.

Propofol induced the infiltration of macrophages from the pro-inflammatory M1 phenotype to the anti-inflammatory M2 phenotype in a rat model of renal ischemia-reperfusion injury (rl/R). The M1 to M2 phenotypes of macrophages were reduced after reperfusion (Liu et al., 2021b), which accelerated the healing of ischemic tissue and promoted the formation of new blood vessels. Propofol may also reduce rl/R-induced acute lung injury (ALI) via the up-regulation of sirtuin (STRT)-1 in the lung, and rl/R stimulation may activate focal proteins caspase-1, nucleotide oligomerization domain (NOD)-like receptor pyrin domain-containing 3 (NLRP3), apoptosis-associated speck-like protein containing a CARD (ASC), IL-1β and IL-18 in the lung, but the expression of STRT1 mRNA and protein were decreased (Liu et al., 2020). STRT1 is an alveolar macrophage inhibitor, and NLRP3 recognizes elevated levels of inflammatory proteins in inflammatory bodies. It is an important stimulus for rl/R to induce ALI (Liu et al., 2018). The protective effect of propofol on organ ischemia-reperfusion and tissue repair may be based on its inhibition of macrophage-involved inflammatory responses and regulation of mitochondrial function.

Propofol attenuates the activation of Kupffer cells during hypoxia-reoxygenation and decreases the expression of TNF-α mRNA by inhibiting [Ca^2+^]i, which attenuates the injury (Sung et al., 2005). The production of TNF-α in Kupffer cells also decreased the activity of caspase-3 and the apoptosis of hepatocytes to alleviate LPS-induced tissue injury and liver dysfunction (Li et al., 2016). Microglia are the resident macrophages of the CNS, and propofol may affect microglia phagocytosis of cell debris. Milk fat globule epidermal growth factor 8 (MFG-E8) is an important regulator of microglial phagocytosis. Cai et al. found that propofol inhibited microglial phagocytosis via the AMP-activated protein kinase (AMPK) and Src signaling pathways regulated by MFG-E8 (Cai et al., 2021b). The activation and proliferation of microglia are typical inflammatory responses to traumatic brain injury (TBI). Several studies suggested that propofol had neuroprotective effects on TBI, which may be due to its inhibitory effect on microglial NADPH oxidase and reduced inflammatory response because of the increased intracranial pressure (Yu et al., 2011; Luo et al., 2013). These findings support a role for propofol in improving brain damage and promoting cognitive recovery after injury by reducing microglial activation and associated neurotoxicity.

#### 4.4.5 Other diseases

Obesity is a metabolic disorder associated with chronic inflammation throughout the body, and adipose tissue is rich in immune cells, including macrophages. An increase in adipose tissue induces chemokine and proinflammatory cytokine (TNF-α, IL-6, Il-12) secretion by monocytes and increases the production and activation of ROS. The resident macrophages in adipose tissue are more likely to differentiate into the pro-inflammatory M1 phenotype (Yu et al., 2011). Propofol may have different immune effects in obese patients than normal-weight patients. A clinical trial that compared general anesthesia with sevoflurane and propofol in the regulation of immune function in patients undergoing laparoscopic weight loss surgery found that propofol differentiated macrophages from adipose tissue to the M2 phenotype (de Sousa et al., 2019). An animal model demonstrated that propofol and lipid excipients had different immunomodulatory effects in obesity, and the active components in propofol increased the expression of IL-10 and M2 phenotypes in macrophages from adipose tissue. Propofol and its active components increased IL-6 expression in obese animals (Heil et al., 2021). With the increase in the obese population, understanding the immune regulation of propofol on macrophages in obese patients will be helpful for proposing an anesthesia management program.

Increased expression of glucose transporter 1 (GLUT1) on the surface of macrophages in adipose tissue suggests that GLUT1 is associated with inflammation *in vivo*. Propofol also regulated the metabolism of macrophages, which regulates the metabolism of activated macrophages by inhibiting the overexpression of ROS and decreasing the expression of GLUT1 (Heil et al., 2021). Arteriosclerosis is a type of inflammatory vascular disease, and the regulation of propofol has a positive effect on arteriosclerosis formation. The expression of connexin 43 (Cx43) in arterial endothelial cells or monocytes induces monocytes to migrate and adhere to the arterial intima then transform into macrophage foam cells, which is a pathological marker for arteriosclerosis. Propofol up-regulates the expression of Cx43 in mononuclear cells and inhibits activation of the PI3K/AKT/NF-κB signaling pathway, which affects cell adhesion and arteriosclerosis formation (Ji et al., 2019).

## 5 Conclusion and outlook

Propofol is widely used in clinical anesthesia. In addition to its sedative and hypnotic effects as an intravenous anesthetic, its characteristics in inflammation, infection, tumor, and tissue injury have attracted much attention in recent years. Macrophages are important immune cells that are involved in inflammation, infection, arteriosclerosis, organ damage and many other diseases. The effect of macrophage activation and polarization on the development of tumors and inflammatory diseases has become a research hotspot. Although some studies demonstrated the effects of propofol on various biological behaviors of macrophages, the mechanisms of propofol regulation of macrophage function are not fully understood. This review focused on the effects and mechanisms of perioperative anesthetics, especially propofol, on the activity and function of macrophages and explored the role of propofol-regulated macrophages in disease progression.

As shown in Table 1 and Figure 2, propofol regulates the role of macrophages in inflammation, infection, tumor, and organ reperfusion injury in cellular and molecular levels. Propofol affects the differentiation and polarization of macrophages in the microenvironment, which leads to different disease outcomes. Propofol inhibits the production of proinflammatory cytokines, which may help to alleviate inflammatory disease and reduce tissue damage. Moreover, it also affects the metabolic activity of macrophages and changes the functional characteristics of macrophages under the original stimulation to make the disease develop to the advantage or disadvantage of the body. The mechanism regulating macrophage function of propofol involves many molecules such as receptors, signaling pathways, epigenetics, and transcription factors. For example, GABAA receptor and TLR4 on the surface of macrophages are the directly or indirectly targets of propofol. Activation of the GABAA receptor pathway, on the one hand, induces the accumulation and nuclear translocation of Nrf2 in the cytoplasm, thereby inhibiting the M1 polarization of macrophages, and ultimately inhibits the secretion of pro-inflammatory cytokines; on the other hand, it inhibits p130cas phosphorylation and further Inhibits phagocytosis of macrophages in response to stress. On the contrary, the expression level of TLR4 is inhibited by propofol, which in turn affects the activation of its downstream NF-κB inflammatory signaling pathway to regulate the immune response. The other involved inflammation-related signaling include MAPK, Foxa2, HNF-α, and AKT. Furthermore, the scorch death pathway NLRP3/ASC/Caspase1 pathway is critical for propofol-induced macrophage pyroptosis. At the epigenetic and transcription factor levels, the expression of some noncoding RNA is regulated by propofol, such as lncRNA LOC286367 and miR-155. However, the mechanism of propofol regulation of transcription and translation and the induction of apoptosis of macrophage-related cytokines at the gene level are not clear and needs further study. Of course, with the deepening of research, the mechanism by which propofol regulates the activation and function of macrophages will become more and more clear, along with the discovery of more regulatory molecules or pathways. In particular, the role of GABA receptors in propofol-regulated macrophages and downstream signaling pathways need to be further investigated. After all, GABA receptor activation has been implicated not only in macrophage phagocytosis, polarization and secretion of inflammatory cytokines, but also in macrophage recruitment, antimicrobial responses and autophagy activation, which is mediated by Ca^2+^, AKT/mTOR, or autophagy-related genes (ATGs) (Kim et al., 2018; Jiang et al., 2019; Bu et al., 2021).

**TABLE 1:** Regulation of propofol on macrophage activation and function.

Macrophage cell types	Propofol dose and time period	Model/Stimuli	Function in macrophages	Related genes	Reference
Murine RAW264.7	50 μM, 16/24 h, *in vitro*	None	↓chemotactic	↓mitochondrial membrane potential; ATP	Wu et al. (2005)
			↓migration		
			↑immunosuppression		
	30 μM, 1/6/4 h, *in vitro*	None	↓migration	↓mitochondrial membrane potential; ATP; INF-γ; ROS	Chen et al. (2003)
			↓oxidant production		
			↓phagocytosis		
	25/50 μmol, 16 h, *in vitro*	LPS	↓inflammation	↓HMGB1; IL-6; IL-8; TNF-α	Jia et al. (2017)
	25/50 mmol/ml, 2 h, *in vitro*	LPS	↓inflammation	↓HMGB1; NF-κB	Wang et al. (2015)
	50 μM, 24 h, *in vitro*	LPS	↓inflammation	↓NO/iNOS; TNF-α; IL-1β; IL-6	Chen et al. (2005)
			↓oxidative		
	<50 μM, 6/12/18/24 h, *in vitro*	LPS	↓inflammation	↓NF-κB; TLR4; TNF-α	Wu et al. (2009)
			↓oxidative		
	6 μg/ml, 5 h, *in vitro*	*s. aureus*	↓phagocytosis	↓IL -1β; ROS	Chen et al. (2020)
	1/5 μg, 24 h, *in vitro*; 20/50 mg/kg, q.h. 3 w, *in vivo*	HCC Model	↑antitumor	↑miR-142-3p	Zhang et al. (2014)
			↓HCC cell invasion	↓RAC1	
	25–200 μg/ml, 24/48/72 h, *in vitro*	None	↑DNA damage	↓DNA-PK; MGMT; BRCA1; p53; MDC1	Wu et al. (2013)
	10 mg/kg, 4 h, *in vivo*	CRC mice	↑intestinal barrier	↓miR-155 NLRP3; TLR4; NF-κB; MPO; TNF; IL-6; IL-1β	Gao et al. (2021)
				↑IL-10	
	10 μg/ml, 0.5 h, *in vitro*	LPS	↓inflammation	↓ROS; AKT/IKKβ/NF-κB; TLR4; NO/iNO_S_; TNF-α; IL-6; IL-10	Hsing et al. (2011)
			↓oxidative		
	25 μg/ml, 24 h, *in vitro*; 10 mg/kg/h, 6 h, *in vivo*	overdose propofol	↑apoptosis	↑GSK-3β	Hsing et al. (2012)
				↓Akt; Mcl	
Peritoneal macrophages (PMs)	100 μM, 30 h, *in vitro*; 10 mg/kg + 5 mg/kg/h, 23 h, *in vivo*	rl/R	↑M2 polarization	↑PPAR-γ; STAT3; IL-10; Arg1mRNA; mrc1mRNA	Liu et al. (2021b)
			↓M1 polarization	↓iNO_S_; TNF-α; IL-6; CXCL-10	
			↓renal I/R injury		
	50 μM, 18 h, *in vitro*; 18.75 mg/kg, *in vivo*	*listeria* monocytogenes (LM) or LPS	↓recruitment	↑IL-1β; IL-6; TNF-α; CCL2; CXCL1; IL-10	Visvabharathy et al. (2015)
			↓activity		
			↑tissue damage		
			↑host susceptibility		
	7.5/15/30 μM, 24 h	LPS	↑immune modulation	↓COX activity; PGE2; IL-12	Inada et al. (2010)
			↓immunosuppression	↑TNF-α	
			↑anti-tumor immunity		
	5 mg/kg, 0.5 h, *in vivo*	LPS	↓inflammation	↓Akt; NF-κB	Hsing et al. (2011)
			↓oxidant	↓NO/iNO_S_	
Human THP-1-cell	1–5 μM, 18 h, *in vitro*	None	↓M1 polarization	↑GABAA; nuclear translocation of Nrf2	Kochiyama et al. (2019)
				↓IL-6; IL-1β	
	2.5–20 μg/ml, 30 min, *in vitro*	20 mmHg pressure	↓phagocytosis	↑GABAA; P130cas	Shiratsuchi et al. (2009)
	50 μM, 24 h, *in vitro*	LPS	↓inflammation	↓RNALOC286267; IL-6; TNF-α; IF-γ	Ma et al. (2018)
				↑ABCA1	
	50 μM, 24 h, *in vitro*	LPS	↓inflammation	↑APOM; HIF-1α	Ma et al. (2013)
				↓TNF-α; IL-1β; IL-6; iNO_S_	
	50/100 μM, 4 h, *in vitro*	LPS	↓glucose metabolism	↓HIF-1α; ATP; LDHA; PDK-1; GLUT1	Tanaka et al. (2010)
				↓VEGF; ENO-1	
Rat alveolar macrophages	50/100 μM, 24 h, *in vitro*	rl/R	↓pyroptosis	↓NLRPS; IL-1β; IL-18	Liu et al. (2020)
			↓acute lung injury	↑STRT1	
Mice bone marrow-derived macrophages (BMDMs)	50 μM, 18 h, *in vitro*; 18.75 mg/kg, *in vivo*	*listeria* monocytogenes (LM) or LPS	↓recruitment	↑IL-1β; IL-6; TNF-α; CCL2; CXCL1; IL-10	Visvabharathy et al. (2015)
			↓activity		
			↑tissue damage		
			↑host susceptibility		
	25/50 μM, 24 h, *in vitro*	LPS	↓inflammation	↓ROS; NADPH; GLUT1	Zeng et al. (2021)
			↓oxidative		
	300/600 μM, 30 min/3/6 h, *in vitro*	None	↑pyroptosis	↑NLRP 3-ASC; caspase-1; ROS	Sun et al. (2019)
				↑IL-1β; IL-18	
Human alveolar macrophages	1.5–2 mg/kg, *in vivo*	100% O_2_ 、30% O_2_	↑phagocytosis	↑IL-1β; IL-8; IFN-γ; TNF-α	Kotani et al. (2000)
			↑microbicidal		
Human peripheral blood mononuclear cells (PBMCs)	0–60 μM, 18 h, *in vitro*	LPS	↓immunosuppression	↓COX-2 activity	Kambara et al. (2009)
				↓PGE2; iNO/NOS; TNF-α; IL-10; TXB2	
Rat Kupffer cell	0.5/5 μg/mg, 5 h, *in vitro*	hypoxia-reoxygenation	↑Kupffer cell activation	↓[Ca^2+^ ]i; TNF-α	Sung et al. (2005)
			↓cell injury		
BV-2 microglia cells	30 μM, 4 h, *in vitro*	LPS	↓neuroinflammation	↓TLR4; GSK-3β; IL-1β; TNF-α; IL-10	Gui et al. (2012)
	12.5/25/50/100 μM, 4 h, *in vitro*	None	↓phagocytosis	↓MFG-E8; AMPK; Src	Cai et al. (2021b)
	25 μg/ml, 24 h, *in vitro*; 10 mg/kg/h, 6 h, *in vivo*	overdose propofol	↑apoptosis	↑GSK-3β	Hsing et al. (2012)
				↓Akt; Mcl	
	12.5–200 μM, 24 h, *in vitro*	TBI, LPS	↑neuroprotective	↓iNO_S;_ NO; TNF-α; IL-1β; ROS; NADPH oxidase; Iba-1	Luo et al. (2013)
			↓microglia activation		
Human microglia	2.5–2 mg/ml, 2/24 h, *in vitro*	15/30 mmHg pressure	↓phagocytosis	↓TNF-α; IL-1β; NO	Yu et al. (2011)
			↓proliferation		

However, propofol-mediated inflammatory diseases and tumors are mostly based on cellular or animal models, but few clinical studies have been conducted in patients. In addition, there are also few studies on the role of propofol in macrophage-associated tumor immunity. One new direction of tumor-targeted immunity is the seeking of new targets using macrophages to phagocytize tumor cells. Understanding the mechanism of macrophage differentiation, proliferation, polarization, apoptosis and phagocytosis is helpful to guide the use of perioperative anesthetics, improve the prognosis and improve the quality of life of patients. Surgical stress-induced immunosuppression is a well-recognized view. Whether the use of propofol inhibits the migration and proliferation of tumor cells by affecting the M1/M2 polarization of macrophages and inhibiting the drug resistance of chemotherapeutic agents to improve the long-term prognosis of tumors requires clarification in the future. To provide a theoretical basis for the development of tumor immunotherapy and related targeted drugs, it is necessary to deepen our understanding of propofol tumor-associated macrophages. The establishment of clinical trials or clinical retrospective investigations will help clarify the accuracy of the study. When propofol is used for general anesthesia and ICU sedation, the effect of propofol on immune cells and oxidative reactions should be considered.

Therefore, more research is needed to elucidate the potential effects of propofol on tumor and inflammation-related disease outcomes by modulating macrophage differentiation and polarization. More clinical studies are needed to confirm the association between perioperative propofol-regulated macrophages and patient morbidity and develop anesthetics with less suppression of immune cell function.

## Glossary

ATG: autophagy-related gene
ALI: acute lung injury
APCs: antigen presenting cells
apoM: apolipoprotein M
AMPK: AMP-activated protein kinase
ASC: apoptosis-associated speck-like protein containing a CARD
ATP: adenosine triphosphate
BMMS: bone marrow-derived macrophages
BRCA1: breast cancer gene 1(BRCA1)
CaMK II: calmodulin-dependent kinase II
CNS: central nervous system
COX: cyclooxygenase
CX43: connexin 43
DNA-Pk: DNA-dependent serine/threonine protein kinase
ERK: extracellular signal-regulated kinase
FoxO1: forkhead box O1
GABA: gamma-aminobutyric acid
GATs: GABA transporters
GLUT1: glucose transporter 1
GSK-3β: glycogen synthase kinase-3
HCC: hepatocellular carcinoma
HIF-1: Hypoxia inducible factor-1
HMGB1: High mobility group box 1
HNF: hepatocyte nuclear factor
ICU: intensive care unit
IFN-γ: interferon-γ
IL: interleukin
iNOs: nitric oxide synthase
lncRNAs: long noncoding RNAs
LPS: lipopolysaccharide
MAPK: mitogen-activated protein kinase
MCAO: middle cerebral artery occlusion
Mcl-1: myeloid cell leukemia 1
MCP-1: chemotactic protein-1
MFG-E8: milk fat globule epidermal growth factor 8
MGMT: O6-methylguanine-DNA methyltransferase
MHC: histocompatibility complex
miRNAs: microRNAs
MMP: matrix metalloproteinase
mTOR: mammalian target of rapamycin
NF-κB: nuclear factor-kappa B
NK: natural killer
NLRP3: nucleotide oligomerization domain (NOD)-like receptor pyrin domain-containing 3
NMDAR: N-methyl-D-aspartate receptor
Nrf2: nuclear factor-E2-related factor 2
PDK-1: pyruvate dehydrogenase (PDH) kinase 1
PGE2: prostaglandin E2
PI3K: phosphatidylinositol 3 kinase
PONV: post-operative nausea and vomiting
PPARγ: peroxisome proliferator-activated receptor gamma
PRIS: propofol infusion syndrome
RAC1: ras-related C3 botulinum toxin substrate 1
rl/R: renal ischemia-reperfusion injury
ROS: reactive oxygen species
STAT: signal transducers and activators of transcriptions
STRT1: sirtuin-1
TAMs: Tumor-associated macrophage
TBI: traumatic brain injury
TLRs: Toll-like receptors
TME: tumor microenvironment
TNF-α: tumor necrosis factors -α
TXA2: thromboxane A2
VCAM-1: vascular cell adhesion molecule-1
VEGF: vascular endothelial growth factor
α2B-ARs: alpha 2-adrenergic receptors
